# Effect of family resilience on subjective well-being in patients with advanced cancer: the chain mediating role of perceived social support and psychological resilience

**DOI:** 10.3389/fpsyg.2023.1222792

**Published:** 2024-04-04

**Authors:** Yating Yang, Fei He, Dongfang Li, Yuanyuan Zhao, Ya Wang, Haoran Zhang, Chan Qiao, Yingfang Cui, Leyun Lin, Hao Guan

**Affiliations:** ^1^School of Public Administration (School of Emergency Management), Northwest University, Xi’an, Shaanxi, China; ^2^Viterbi School of Engineering, University of Southern California, Los Angeles, CA, United States; ^3^Department of Burns and Cutaneous Surgery, Xijing Hospital, Xi’an, China

**Keywords:** patients with advanced cancer, subjective well-being, perceived social support, psychological resilience, quality of life

## Abstract

**Purposes:**

Domestic and international research has found that patients with advanced cancer prioritize increasing their quality of life above extending their lives with simple or intensive treatments. The current study investigates the pathways to improve patients’ sense of well-being from the family, social, and individual levels, that is to say, it investigates the mediating roles of comprehending social support as well as psychological resilience in the relationship between family resilience and subjective well-being, and it also provides references for future intervention.

**Method:**

The Family Resilience Questionnaire (FRQ), General Well-being Schedule (GWB), Perceived Social Support Scale (PSSS), and the Chinese version of the Cornor-Davidson Resilience Scale 10-item (CD-RISC) were all completed by 338 patients with advanced cancer who took part in the study.

**Results:**

The study’s findings demonstrated a significant and positive correlation between family resilience, subjective well-being, perceived social support, and psychological resilience. Additionally, there was a significant direct effect of family resilience on subjective well-being as well as a mediating and chain mediating effect between perceived social support and psychological resilience. The findings of this study will be very helpful in the future when it comes to enhancing the quality of life for patients with advanced cancer through intervention.

**Conclusion:**

Subjective well-being can be influenced directly by the family resilience of advanced cancer patients, or indirectly through the psychological resilience and perceived social support.

## 1 Introduction

In 2020, there were approximately 19.3 million new instances of cancer worldwide, according to the Global Cancer Data Report (Sung et al., 2021). China saw 4.82 million new cases of cancer in 2022 (Xia et al., 2022). Due to the traumatic nature of cancer, patients may experience a range of negative feelings following a diagnosis, including anxiety, sadness, psychological distress, and so forth. These effects can have a major detrimental impact on a patient’s quality of life (QoL) (Cardoso et al., 2015). Enhancing the quality of life for cancer patients has emerged as a significant public health issue that medical professionals around the world must deal with. In an effort to discover more effective strategies to enhance the quality of life for cancer patients, numerous research have been carried out to investigate the variables influencing the mental health of these patients as well as the underlying mechanisms.

In recent years, more and more studies have discovered that, in addition to negative psychological changes, individuals also have some positive changes, that is, positive psychological changes after encountering stressful situations (Cao et al., 2017). It encourages people to have an optimistic outlook in order to see psychological issues and occurrences from a fresh perspective and to develop their own good qualities and abilities, whether they are present now or not (Jiang and Peng, 2017). As a component of positive psychology, subjective well-being is a comprehensive psychological criterion for assessing life satisfaction (Yan, 2003). Research has demonstrated that cancer patients’ subjective well-being positively affects their quality of life (Edelstein et al., 2015). Subjective and objective elements are the two categories into which the influencing factors of the subjective well-being of cancer patients can be separated. According to Han et al. (2018), the objective factors include primarily social support, physical health status, economic situations, and demographics. The subjective aspects are coping styles, psychological distress, personality traits, and self-esteem.

Previous research has not thoroughly investigated the effects of individual psychological factors, social factors, and familial factors on subjective well-being, nor have they explored the relationships between them. Instead, it concentrated on looking at a particular aspect of subjective well-being. Therefore, based on the perspective of positive psychology, this study examines the internal mechanisms and family, social, and individual psychological factors that influence cancer patients’ subjective well-being. It also provides a theoretical framework for achieving improvements in cancer patients’ quality of life and physical and mental health.

In conclusion, the purpose of this study was to investigate the connection between Chinese advanced cancer patients’ subjective well-being and family resilience. It also looked at how psychological resilience and perceived social support might act as moderators.

## 2 Background

### 2.1 Family resilience and subjective well-being

Patients and their family experience profound emotional distress upon receiving a cancer diagnosis, particularly if the disease has gone to an advanced stage and there is no chance of recovery. As a patient approaches the end of their life, the goal of treatment shifts from saving life to maximizing comfort and upholding the patient’s dignity in a larger sense (World Health Organization, 2002; Muñiz and Pérez, 2009). Cancer patients experience physical, psychological, and mental discomfort at the same time as other confrontations. This can lead to psychological issues like depression, anxiety, despair, and loneliness, which lowers the patients’ subjective well-being. Additionally, the constant struggle these patients endure during their illnesses has a significant negative impact on their quality of life (Lutgendorf et al., 2017).

Subjective well-being (SWB) is a concept that first appeared in positive psychology and health psychology. It is a comprehensive index that assesses an individual’s total quality of life and represents how satisfied certain groups are with their living situations. It encompasses both positive and negative subjective experiences and an assessment of life satisfaction. Subjective well-being increases with an individual’s overall life satisfaction and the amount of good feelings they experience (Yennurajalingam et al., 2017). According to previous studies, factors that influence subjective well-being to varying degrees include illness factors (Edelstein et al., 2015), social support (McDonough et al., 2013), and self-esteem (Devins et al., 2015). However, recent research has discovered a strong relationship between subjective well-being and psychological resilience (Wang and Chen, 2018), perceived social support (Jiao, 2020), and family resilience (Wang et al., 2021).

The concept of family resilience (FRS) was first put forth by McCubbin and McCubbin (1988), and it belongs to one of the functions of the family (Wu et al., 2018). This function pertains to the ability of the family as a whole to overcome and cope with challenges and difficulties together, as well as to recover from stresses and crises and gain additional psychosocial resources (Walsh, 1996). A previous study investigated families of children with cancer and found that resilience factors contributing to family adjustment included family resilience, problem solving and coping, social support, and model of family functioning (Sim, 2004); Wang et al. (2015) also apply McCubbin’s family resilience model to the adjustment of cancer patients’ families, and use the family resilience model as an entry point for nursing assessment, in order to increase family confidence in patients’ care, strengthen family functions, and enable families to tend to a positive process of adjustment. Studies have also shown that family members who experience positive family functioning are more inclined to practice self-care activities, like taking their medications and getting exercise (Shamali et al., 2019; Yeom and Lee, 2020). Furthermore, it fosters efficient communication, problem-solving skills, and an increased capacity to overcome obstacles, all of which positively affect patients’ and their families’ physical and mental health (Panyavin et al., 2015) and enhance their quality of life (Kołtuniuk et al., 2019). Currently, Taiwan is the site of successful exploratory applied studies utilizing the family resilience approach in the areas of depression, traumatized families, rare disease patients, and older people with impairments.

Although many researchers have examined the relationship between the family function and quality of life in children (Hocking et al., 2011, 2015) and cancer survivors (Lim and Ashing-Giwa, 2013), there is currently a dearth of research on the relationship between family resilience and subjective well-being in patients with advanced cancer. Good family functioning and family support can reduce the stress of illness, provide cancer patients with emotional support, and improve their quality of life (Cardoso et al., 2015). This is because families are important external influences on subjective well-being and are one of the most important systems of support for patients with advanced cancer (Kossek et al., 2018). As a family strength, family resilience provides significant advantages for advancing both individual and family health. Studying its connection to patients’ subjective well-being who have advanced cancer is therefore essential.

### 2.2 The mediating role of psychological resilience

More research is necessary to determine the psychological mechanisms that may influence subjective well-being through family resilience; psychological resilience may act as a mediating factor in this relationship. Today, the majority of research defines psychological resilience as a person’s ability to bounce back psychologically from obstacles and failures. According to Luthar et al. (2000), this process is dynamic and regular, and it expresses itself as a person’s inner condition throughout trying times. Though well-adjusted individuals have not been the subject of many studies on the psychology of cancer patients, mental toughness should be taken into consideration when examining and evaluating the psychological states of these individuals, as they tend to be positively psychologically state. Well-adjusted cancer patients can effectively manage stress, exhibit positive and optimistic attitudes toward life, and obtain longer survival and higher quality of life (Shen and Wang, 2006). Some studies have shown that positive attitudes are significantly associated with patients’ mental status and quality of life (Wang et al., 2010a). The psychological resilience of cancer patients has been examined by other studies in relation to psychological status, individual characteristics, family resources, social support, and various cancer kinds and stages of the disease. For instance, Beasley et al. (2003) contend that psychological resilience helps cancer patients feel less uncomfortable psychologically and that it helps people feel less anxious and depressed as well as more confident and hopeful when faced with obstacles in life. In the same way that Pentz (2005) discovered that social support and spiritual beliefs play a significant role in the psychological resilience of elderly cancer patients, West et al. (2012) demonstrated the positive effects of a supportive family environment on psychological resilience. As a result, considering cancer patients’ psychological states from the standpoint of psychological resilience has important practical implications for their mental health.

According to the bio-social-cognitive theory model, external environmental elements can have an indirect impact on subjective well-being in addition to their direct influence. Subjective well-being is the outcome of the interaction between external environmental factors and interior cognitive factors. Furthermore, internal cognitive processes may have an indirect impact on subjective well-being (Lyons et al., 2013). Therefore, as internal cognitive elements, perceived social support, self-esteem and psychological resilience may also be essential determinants determining subjective well-being. Furthermore, Haase et al.’s (2014) illness psychological resilience model indicates that family dynamics play a significant protective role in cancer patients’ psychological resilience. Effective family function can help cancer patients become more psychologically resilient, which lowers their personal maladaptive responses (Kim et al., 2018). Good psychological resilience is beneficial to the psychological health and recovery of cancer patients (Sisto et al., 2019). Li et al. (2017) state that there is a significant positive correlation between an individual’s psychological resilience and the family’s resilience. The majority of research has also demonstrated that psychological resilience positively predicts subjective well-being and is a significant internal component of subjective well-being (Wang et al., 2021). Thus, the impact of familial resilience on subjective well-being may be mediated by psychological resilience.

### 2.3 The mediating role of perceived social support

In addition to the effects of family resilience and psychological resilience on subjective well-being, perceived social support is also closely related to subjective well-being. The resources that people obtain from social interactions, such as financial or spiritual support from friends and family, are referred to as social support (Cullen, 1994). One of the key factors influencing people’s ability to adjust to new social circumstances is social support, which can serve as a buffer when people experience negative events (Cohen and Wills, 1985). In general, social support can be separated into two groups: perceived and actual social support, or objective and subjective support. Subjective support, also known as perceived social support, is the emotional state in which people feel valued, understood, and supported; objective support, or actual social support, comprises financial aid and direct services that people receive (Xiao and Yang, 1987; Li, 1998). Cancer patients’ quality of life is strongly correlated with their perceived social support status. Improving their perceived social support can help patients feel more respected, cared for, and supported emotionally, as well as enhance their own coping mechanisms with the illness (Krajmer et al., 2017; Pasek et al., 2017).

Perceived social support is more likely to demonstrate an individual’s ability to improve their mental health than factual social support (Niu, 2014). Numerous studies have demonstrated that cancer patients’ psychological resilience increases with their perceived level of social support (Somasundaram and Devamani, 2016; Costa et al., 2017). He et al. (2015), for instance, found that social support had the most predictive power for psychological resilience in a study of 200 cancer treatment patients. Perceived social support refers to the individual’s emotional experience of being respected, understood and supported in the subjective sense, which is a process of cognitive understanding. It focuses more on the expectations and evaluation of the social support received by individuals (Ye, 2006). When encountering difficulties or setbacks and getting practical help from others, whether an individual is aware of, and how to interpret, this actual helping behavior is more important in protecting their subjective well-being (Lian, 2017). According to social support theory, people are more likely to report higher levels of subjective well-being and to feel more confident in their ability to handle challenges or setbacks when they have a higher perceived social support system (Liu and Cheng, 2019). Empirical studies have also found that perceived social support significantly predicts subjective well-being (Wang and Zhang, 2019). Thus, it is believed that one of the mediators of subjective well-being is perceived social support.

### 2.4 The present study

Based on family resilience theory (McCubbin and McCubbin, 1988), family strengths must be used in interactions at all societal and cultural levels in order to maximize the power of family resilience. According to Walsh (2012) and Das et al. (2017), there is a positive link between family resilience and perceived social support in previous studies. Patients who possess family resilience are better able to utilize social support systems. Furthermore, it has been shown that psychological resilience and perceived social support are positively correlated, and that those who have high psychological resilience are also better able to cope with adversity (Zhong et al., 2019). The mediating function of psychological resilience in perceived social support and subjective well-being was also supported by a study on the subjective well-being of “The old drifters” (Jiao, 2020). This implies that the subjective well-being of patients with advanced cancer may be enhanced by perceived social support and psychological resilience, which are protective factors that are effective.

**Figure 1:**
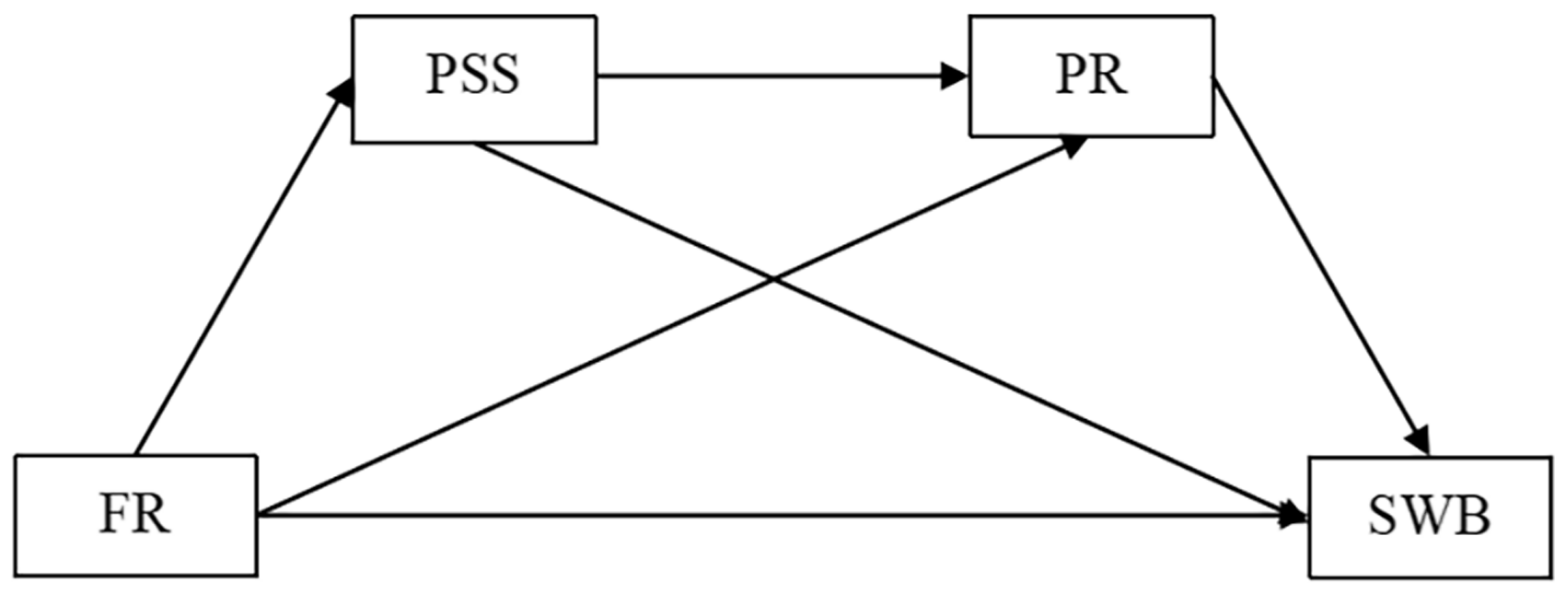
The proposed chain mediation model. PSS, perceived social support; FR, family resilience; SWB, subjective well being; PR, psychological resilience.

Consequently, we proposed the following hypotheses (see Figure 1):

Hypothesis 1: Family resilience will affect subjective well-being through perceived social support.

Hypothesis 2: Family resilience will affect subjective well-being through psychological resilience.

Hypothesis 3: Perceived social support and psychological resilience will play a chain mediating role in the effect of family resilience on subjective well-being.

## 3 Materials and methods

### 3.1 Participants

The participants were inpatients at a specialized oncology hospital in Shanxi Province, China. Patients were recruited from April 2nd to April 16th, 2022. The participants were given paper-based, anonymous questionnaires in the wards. At the end of the study, patients could choose their favorite gift to take away among the gifts the researchers had prepared beforehand.

Inclusion criteria: (1) Those diagnosed with stage III or IV cancer by pathological diagnosis; (2) Those with condition permitting, informed consent and able to cooperate.

Exclusion criteria: (1) Patients with recurrence, metastasis or other serious complications; (2) Patients with communication difficulties, unconsciousness, and previous history of psychological disorders and psychiatric disorders; (3) Patients who have not reached the statutory age of adulthood and whose family members do not wish to participate in the study.

### 3.2 Data

A questionnaire survey was conducted using the convenience sampling approach. The sample size for this study is obtained by multiplying the number of questionnaire questions by 5–10. Since all questionnaire questions in this study are 60, the total number of subjects is 300–600. According to the inclusion criteria, 378 questionnaires were disseminated, 378 questionnaires were collected, 40 invalid questionnaires were removed (missing data >5% and outliers), 338 valid questionnaires were retrieved, and the effective rate was 89.4%.

### 3.3 Procedure

The Northwest University Ethics Committee granted its approval for this project. Informed consent was provided for all participants reached the legal age. For participants under the legal age, consent was obtained from their legal guardians. All participants were informed of the importance of the truthfulness and completeness of their responses. The anonymity of this study was emphasized prior to data collection.

### 3.4 Measures

#### 3.4.1 Family resilience

The Family Resilience Questionnaire (FRQ), developed by Bu and Liu (2019), was used to examine the ability to recover and grow from adversity. The scale consisted of 20 items with four dimensions of perseverance, support, rapport and openness. A 7-point scale was used to calculate the total score for all items, with higher scores associated with better levels of family resilience. In this study, the Cronbach’s α indicating reliability was 0.97, and the KMO value indicating validity was 0.97.

#### 3.4.2 Subjective well-being

The General Well-being Schedule (GWB), developed by Fazio (1977) of the National Center for Health Statistics and revised by our scholar Duan (1996), was used to measure the level of general well-being in the most recent month. The scale consists of 18 items with 6 dimensions: satisfaction and interest in life, worry about health, energy, depressed or pleasant state of mind, control over emotions and behaviors, and relaxation and tension. A 5-point scale was used to calculate the total score for all items, with higher total scores indicating greater subjective well-being. In this study, the Cronbach’s α indicating reliability was 0.93, and the KMO value indicating validity was 0.94.

#### 3.4.3 Perceived social support

The Perceived Social Support Scale (PSSS), which was developed by Zimet et al. (1988) and translated and revised by Jiang (2001), is a 12-item scale consisting of three subscales: family support, friend support, and other support (teachers, classmates, and relatives). Each subscale consists of four items. A 7-point scale was used to calculate the total score of all indicators, and the higher the score, the higher the total social support level obtained. In this study, the Cronbach’s α indicating reliability was 0.96, and the KMO value indicating validity was 0.95.

#### 3.4.4 Psychological resilience

The modified Chinese version of the Cornor-Davidson Resilience Scale 10-item (CD-RISC 10), by Wang et al. (2010b) was used, which is suitable for the study of cancer patients and is a unidimensional scale with 10 items, using a 5-point scale to calculate the total score of all items, and the higher the score, the higher the patient’s psychological the higher the score, the higher the patient’s level of psychological resilience. In this study, the Cronbach’s α indicating reliability was 0.95, and the KMO value indicating validity was 0.95.

### 3.5 Data analysis

The data was entered using the SPSS25.0 software, and descriptive statistics as well as correlation analysis were performed. Model 6 of the SPSS macro program PROCESS v4.1, created by Hayes, was used to conduct the mediating effect analysis, which controlled for age, education, and monthly family income. It examined the role that psychological resilience and perceived social support played in mediating the relationship between subjective well-being and family resilience, and it used the deviation correction of the percentile Bootstrap method for the mediation effect test.

The PROCESS plug-in is specifically designed for analyzing mediating and moderating effects, making it compatible with popular statistical analysis software such as SPSS and SAS. In addition to the visual operations available in SPSS, users can also utilize Syntax and other methods to enhance functionality. With over 90 models available, selecting the appropriate model and specifying independent variables, dependent variables, mediators or moderators is all that’s required for analysis using PROCESS. Unlike traditional SPSS which necessitates stepwise or hierarchical regression for mediating and moderating effects, PROCESS accomplishes this in a single step while automatically processing Bootstrap and Sobel tests.

We can more precisely estimate *p*-values for hypothesis tests and eliminate bias in the estimates by utilizing the bias-corrected percentile bootstrap approach. The bias-corrected percentile bootstrap method is an excellent statistical tool for calculating confidence intervals for parameters and hypothesis testing. It allows us to estimate the parameters more correctly and reduces estimation bias, and the Sobel test is essentially a specialized t-test used to examine whether the inclusion of the mediator variable in the model significantly reduces the influence of the independent variable, and hence whether the mediating effect is statistically significant.

## 4 Results

### 4.1 Descriptive statistics

The age of the subjects in this study ranged from 11 to 87 years, with a mean age of 49.20 ± 15.10 years, 61.8% were female and 84.6% were married, as shown in Table 1.

**Table 1:** Descriptive statistics of variables.

Variables		Frequency (*N*)	Percentage (%)
Age	11–87	338	
Gender	Male	129	38.2
	Female	209	61.8
Education	Primary school and below	54	16.0
	Junior high school	91	26.9
	High school or technical secondary school	89	26.3
	Junior college	62	18.3
	Bachelor degree or above	42	12.4
Marital status	Unmarried	37	10.9
	Married	286	84.6
	Divorced	8	2.4
	Widowed	7	2.1
Occupation	Farmer	185	54.7
	Worker	36	10.7
	Public institution	50	14.8
	Self-employed	26	7.7
	Retirement	12	3.6
	Other	29	8.6
MFI	Below2000	98	29.0
	2000–5,000	194	57.4
	Above 5,000	46	13.6
TOHI	BMIFE	96	28.4
	BMIFUR	231	68.3
	PBSMU	2	0.6
	Other medical insurance	2	0.6
	No insurance	7	2.1

*N* = 338. MFI, monthly family income; TOHI, type of health insurance; BMIFE, basic medical insurance for employees; BMIFUR, basic medical insurance for urban and rural residents; PBSMU, policy-based supplementary medical insurance.

### 4.2 Correlation analysis of family resilience, subjective well-being, perceived social support and psychological resilience

The correlation analysis of family resilience, subjective well-being, perceived social support and psychological resilience shows that family resilience, subjective well-being, perceived social support and psychological resilience are significantly positively correlated (as shown in Table 2).

**Table 2:** Descriptive statistical results and correlation analysis between variables.

	*M*	SD	1	2	3	4
1. Family resilience	110.30	20.97	1			
2. Subjective well-being	65.38	12.91	0.785**	1		
3. perceived social support	28.99	7.47	0.631**	0.641**	1	
4. psychological resilience	63.58	11.87	0.449**	0.464**	0.441**	1

*N* = 338. *p < 0.05, **p < 0.01.

### 4.3 Model validation

The PROCESS regression analysis revealed (as shown in Table 3) that family resilience was a significant direct predictor of subjective well-being in patients with advanced cancer (*β* = 0.602, *p* < 0.001); the level of family resilience was a direct positive predictor of perceived social support (*β* = 0.607, *p* < 0.001) and psychological resilience (*β* = 0.272, *p* < 0.001); perceived social support was a direct positive predictor of psychological resilience (*β* = 0.262, *p* < 0.001) and subjective well-being (*β* = 0.210, *p* < 0.001); and psychological resilience positively predicted subjective well-being in patients with advanced cancer (*β* = 0.099, *p* < 0.01).

**Table 3:** Regression analysis of variable relationships in chained mediation models.

Regression equation		Overall fit index			Regression coefficient significance			
DV	IV	*R*	*R* ^2^	*F*	*B*	β	*t*	VIF
PSS	FR	0.656	0.430	62.825***	0.216	0.607	14.395***	1.040
	Age				0.073	0.148	2.956**	1.471
	MFI				−1.329	−0.113	−2.637**	1.074
	Education				1.112	0.187	3.703***	1.483
PR	FR	0.501	0.251	22.261***	0.154	0.272	4.409***	1.687
	PSS				0.417	0.262	4.170***	1.755
	Age				−0.042	−0.053	−0.909	1.509
	MFI				−1.276	−0.068	−1.374	1.096
	Education				−0.343	−0.036	−0.614	1.544
SWB	FR	0.816	0.665	109.685***	0.371	0.602	14.173***	1.785
	PSS				0.363	0.210	4.861***	1.847
	PR				0.107	0.099	2.683**	1.335
	Age				−0.024	−0.029	−0.728	1.513
	MFI				0.324	0.016	0.478	1.103
	Education				0.665	0.065	1.632	1.545

*N* = 338. DV, dependent variable; IV, independent variable; MFI, monthly family income; PSS, perceived social support; FR, family resilience; PR, psychological resilience; SWB, subjective well-being. *p < 0.05, **p < 0.01, ***p < 0.001.

Table 4 displays the findings of the Bootstrap method test for mediating effects with 5,000 repeated samples. The results presented in Table 4 demonstrate the significant direct impact of family resilience on subjective well-being, as well as the noteworthy mediating effects of perceived social support and psychological resilience. Furthermore, a chain mediating effect is observed between perceived social support and psychological resilience. Hypothesis 1 was confirmed when the effect of perceived social support partially mediated the relationship between family resilience and subjective well-being, with an effective value of 0.128, accounting for 26.947% of the total effect value; hypothesis 2 was confirmed when the effect of psychological resilience partially mediated the relationship between family resilience and subjective well-being, with an effective value of 0.027, accounting for 5.684% of the total effect value; and hypothesis 3 was confirmed when perceived social support and psychological resilience played a chain mediating role between family resilience and subjective well-being, with an effective value of 0.016, accounting for 3.368% of the total effect value.

**Table 4:** Mediating effects of perceived social support and resilience.

Paths	Path Effect	Effect size(%)	95% CI	
			Lower limit	Upper limit
FR – PSS – SWB	0.128	26.947	0.057	0.196
FR – PR – SWB	0.027	5.684	0.008	0.053
FR – PSS – PR – SWB	0.016	3.368	0.004	0.031
Direct effect	0.371	78.105	0.319	0.422
Total effect	0.170	35.789	0.100	0.241

*N* = 338. PSS, perceived social support; FR, family resilience; PR, psychological resilience; SWB, subjective well-being.

**Figure 2:**
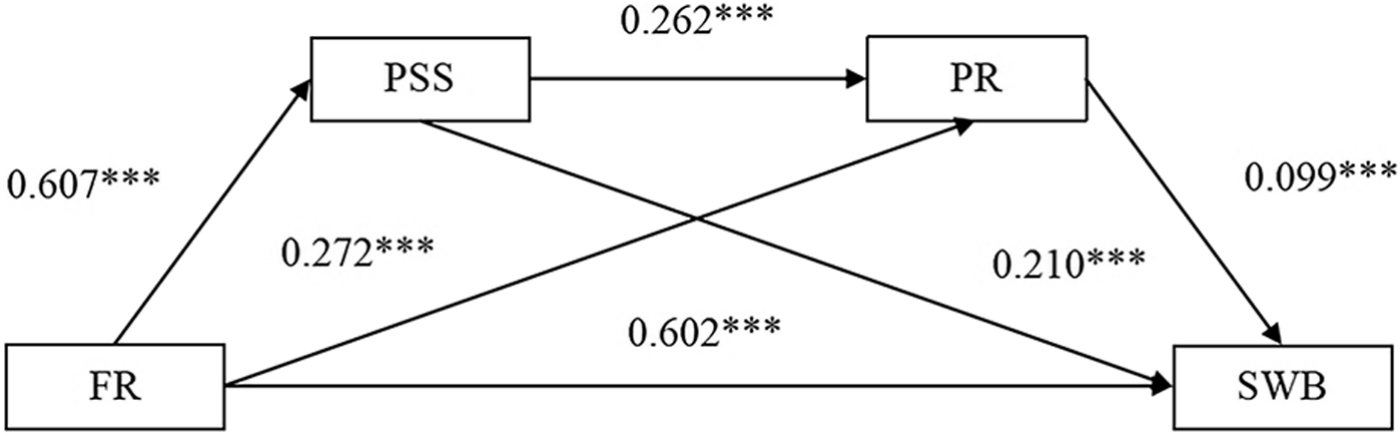
The chain mediating role of perceived social support and psychological resilience. PSS, perceived social support; FR, family resilience; PR, psychological resilience; SWB, subjective well-being. *P < 0.05, **p < 0.01, ***p < 0.001.

In conclusion, the results of this study validated the initially proposed hypothesis model (see Figure 2).

## 5 Discussion

### 5.1 Relationship between family resilience and subjective well-being in patients with advanced cancer

This study first investigated the association between family resilience and subjective well-being and discovered that family resilience significantly predicted the subjective well-being of patients with advanced cancer, which is consistent with prior research (Yang and Zhang, 2021).

According to the findings of this study, patients’ subjective well-being may be associated to family resilience. The reasons for this can be analyzed as follows: cancer primarily affects the elderly population, patients frequently face increased psychological stress, the onset of the disease causes physical pain, and the recovery period may cause patients to worry about the disease’s prognosis; Because of the unique characteristics of advanced cancer, clinical therapy is more difficult, and patients require long-term medicine, which causes mental stress and induces negative feelings. Patients with low family resilience lack the care and encouragement of family members and face challenges that are difficult to resolve in a timely manner, resulting in a pessimistic mentality that impacts persons’ subjective feeling of well-being to some extent. Higher family resilience can foster a sense of emotional responsibility, assist patients in reducing psychological stress, and urge patients to mobilize internal psychological resources to better control their personal emotions through counsel and support. Furthermore, when completing functional rehabilitation exercises, good family resilience may share the hardship and agony of patients, modify beneficial changes in patients’ behaviors, which is favorable to improving patients’ negative emotions and subjective well-being.

### 5.2 Mechanisms of family resilience and subjective well-being in patients with advanced cancer

The findings revealed a significant positive correlation among family resilience, perceived social support, psychological resilience, and subjective well-being, which is consistent with previous research results (Liu and Xu, 2015; Das et al., 2017; Li et al., 2017; He et al., 2018; Liu and Bu, 2020; Wang et al., 2020). This study showed that perceived social support mediates the relationship between family resilience and subjective well-being. This implies that family resilience influences subjective well-being through perceived social support, with the greatest amount of mediating effect occurring along the perceived social support mediating path. This could be because cancer is a really bad life event that affects people individually, in families, and in society as a whole. It causes people to suffer physically and mentally and puts their family through a lot. Patients with high levels of family resilience can positively tackle this terrible occurrence and increase their subjective well-being, as families are the primary caretakers for patients with advanced cancer in China (Sim, 2004). Furthermore, the primary social support system for people with advanced cancer is their family. Patients who have strong family resilience are better able to take advantage of social support, feel supportive from others, and improve their own coping skills. These factors have a significant influence on patients’ positive disease-related coping, which in turn raises their subjective well-being.

Moreover, psychological resilience acts as a moderator between subjective well-being and family resilience, meaning that psychological resilience enables family resilience to affect subjective well-being. This corresponds with the discoveries made by Wang et al. (2021). According to Greeff et al. (2014), families that show high levels of family resilience are also better at buffering and handling stressful situations. Patients’ perceptions of family cohesiveness, intimacy, and open communication, in turn, support higher levels of psychological resilience. When faced with disease stress, advanced cancer patients with higher levels of psychological resilience are able to invoke more psychological resilience factors (protective factors) to quickly adjust their mindset, positively adapt to and face the disease, and increase their confidence in disease treatment, reducing their level of despair. According to Antony et al. (2018), families that exhibit strong family resilience are more adaptive, provide their patients with sufficient medical treatment and financial assistance, and improve their patients’ subjective well-being. High psychological resilience people are adept at controlling their emotions, adjusting to their surroundings, changing their perspective when faced with challenges, and handling crises. This enhances their subjective and psychological well-being.

Finally, this study found a significant correlation between perceived social support and psychological resilience and family resilience and subjective well-being. This could be because patients’ family resilience improves their perceived social support, patients’ perceived social support improves their ability to cope with difficult situations, which increases their psychological resilience, and individuals with high levels of psychological resilience actively adjust, increasing their subjective well-being. Patients with high levels of family resilience are able to make greater use of social support networks, which aids in the relief of stress and negative emotions; when people sense the support of family, friends, and society, they are more motivated to seek solutions to issues. In other words, social support can provide spiritual benefits and increase a person’s sense of well-being. In conclusion, perceived social support and psychological resilience are both internal and external protective variables that enhance individual life quality improvement. It is critical in the process of enhancing subjective well-being.

Subjective well-being greatly impacts patients’ quality of life. Patients’ psychological states will alter dynamically with the progression of the disease and changes in treatment as the incidence of cancer rises. It is critical to pay attention to the dynamic changes in subjective well-being of cancer patients at various stages, as well as the effects of various interventions on them. This study initially revealed the importance of perceived social support and psychological resilience in mediating the relationship between family resilience and subjective well-being. Cancer patients should be treated with respect, kindness, care, and support from their families and society in a variety of methods, with a focus on the positive role of individual psychological resilience. To increase the patients’ mental toughness by psychological intervention and to construct the intervention program from the family’s standpoint, When caring for patients with advanced cancer, it is suggested that patients’ psychological resilience be improved by enhancing protective factors, encouraging family visits and companionship, establishing community service groups, increasing collaboration between nurses and patients, and sharing experiences among patients. To improve the patients’ subjective well-being and, as a result, their quality of life.

This study establishes the theoretical basis for improving the subjective well-being of advanced cancer patients. It broadens the scope of positive psychology research, supports the shift of the center of care model from the patient to the family and society, and serves as an instance for the psychological rehabilitation of families with advanced cancer patients in China. In practice, it assists advanced cancer patients and their families in discovering the importance of family social strengths as well as individual psychological resources, establishing an internal family support system, positively coping with challenges, enabling the family to successfully overcome the crisis, and promoting the patient’s and family’s recovery.

## 6 Limitations

Some limitations should be considered when evaluating the study’s findings:

First, the subjects in this study were only hospitals from the same city, which may be underrepresented; future studies can investigate diverse locations and cancer types.

Second, all factors were analyzed using self-report, which may have affected the validity of this study; future studies might be evaluated by incorporating the evaluation of family members as well as doctors and nurses.

Furthermore, this study only looked at the route analysis model with explicit manifest variables, not the structural equation model with latent variables.

Finally, due to time limits, this study used a cross-sectional survey, which may have the drawback of restricted strength of inference for the interrelationships among components, and did not undertake an in-depth investigation and follow-up intervention. Future research could investigate interventions in family resilience, psychological resilience, or perceived social support in advanced cancer patients using a combination of cross-sectional and longitudinal research designs, quantitative and qualitative research methods, and randomized trial studies. Interventions could, for example, be carried out through group counseling activities such as mindfulness or cognitive behavioral therapy.

Nonetheless, despite its limitations, this study makes some theoretical and practical contributions. This study, from a theoretical standpoint, extends earlier research by illustrating the chain mediating role of perceived social support and psychological resilience. According on the findings of this study, interventions at the family, social, and individual levels can be implemented in the future. Nursing staff can provide regular training and counseling to patients’ families and patients themselves, and patients’ families can also communicate with patients more frequently to understand patients’ emotions and thoughts, thereby improving the quality of life of patients with advanced cancer and extending survival time in many ways.

## 7 Conclusion

The present study investigated patients with advanced cancer and included perceived social support and psychological resilience into the analysis to explore their mediating role between family resilience and subjective well-being. The analysis of the mediation effect revealed that, family resilience of patients with advanced cancer might not only affect the level of subjective well-being of cancer patients directly, but also indirectly by perceived social support and psychological resilience as mediating variables. The mediation model included three mediation paths: (1) the mediation path through psychological resilience; (2) the mediation path through perceived social support; and (3) the mediation path through both psychological and perceived social support. The results of this investigation correspond with our theoretical frameworks and help to improve the quality of life for cancer patients.

This study was reported according to the STROBE guideline.

## Data availability statement

The raw data supporting the conclusions of this article will be made available by the authors, without undue reservation.

## Ethics statement

This study was approved by the Ethics Committee of Northwest University. Informed consent was provided for all participants over the age of majority. For participants under the legal age, consent was obtained from their legal guardians.

## Author contributions

YY, FH, and DL: conceptualization, investigation, and formal analysis. YY: writing - original draft. FH: validation, supervision, and writing - review and editing. YY and DL: methodology. YZ, HZ, and CQ: Visualization. YW: resources and software. YY and FH: validation, supervision, and writing - review and editing. YZ, YC, and LL: data curation. HG and FH: funding acquisition.
